# Epidemiology and treatment of 23 musicians with task specific tremor

**DOI:** 10.1186/2054-7072-1-5

**Published:** 2014-12-04

**Authors:** André Lee, Shinichi Furuya, Eckart Altenmüller

**Affiliations:** Inistitute of Music Physiology and Musicians’ Medicine, Hannover University of Music, Drama and Media, Hannover, Germany

**Keywords:** Dystonia, Essential tremor, Movement disorders, Botulinum toxin, Deep brain stimulation

## Abstract

**Background:**

Task specific tremors in musicians have been mainly described as primary bowing tremor in string instrumentalists in relatively small sample sizes. Our aim was to describe epidemiology, risk factors, phenomenology and treatment options of this disorder in 23 musicians of different instruments.

**Methods:**

We included 23 professional musicians (4 female, 19 male; mean age 51.5 ± 11.4 years) with a TSTM. During anamnesis, clinical examination, by mail or via telephone patients were asked for epidemiological, phenomenological information, risk factors and treatments. We then compared our findings to primary writing tremor, the most common task specific tremor.

**Results:**

Age at onset of the TST was 44.6 ± 13.6 years and tremor appeared 35.1 ± 13.5 years after beginning to play the instrument. The majority of patients were string instrumentalists, followed by woodwind instrumentalists. Other instrumentalists were a guitarist, pianist and percussionist respectively. In contrast to primary writing tremor, we also found proximal muscles of the upper extremity involved in tremor. A positive family history was found in Prior trauma was more common than in primary writing tremor. Treatment with a positive effect on tremor were in order of efficacy: Botulinumtoxin, Primidone, Propranolol, Trihexyphenidyl. No patient had undergone deep brain stimulation.

**Conclusion:**

Task specific tremor in musicians is a heterogeneous disorder with a male gender predominance that shares many commonalities with PWT. The onset age as well as the time between starting to play the instrument and tremor onset has a wide range. Because previous trauma and overuse appear to be risk factors, preventive measures against playing related injuries are necessary. There appears to be a genetic predisposition for TST. No single beneficial medication exists and treatment of patients remains highly individual. It should be discussed, whether deep brain stimulation should be offered not only to patients that do not respond to any other medication but early in the course of the disease.

**Electronic supplementary material:**

The online version of this article (doi:10.1186/2054-7072-1-5) contains supplementary material, which is available to authorized users.

## Background

Task specific tremor in musicians (TSTM) is a highly disabling disorder that occurs only or mainly while playing the instrument and has primarily been described in string instrumentalists as primary bowing tremor (PBT) [1–3]. The most common form of a task specific tremor is primary writing tremor (PWT), first described by Rothwell in 1979 [4]. Two forms can be distinguished: type A tremor (task specific) and type B (position specific) [5]. While there are many studies on epidemiology, pathophysiology and treatment of PWT [5–20] only a few studies are available for TSTM [1–3, 21]. Thus the sample sizes described so far are small, allowing only for limited conclusions e.g. with regard to risk factors or treatment. The aim of this paper therefore was to describe epidemiological data and treatment in 23 musicians with TSTM at a variety of instruments and compare the findings to PBT and PWT.

## Methods

The study was approved by the local ethics committee and written informed consent was obtained from all participants.

We included 23 professional musicians (4 female, 19 male; mean age 51.5 ± 11.4 years) with a TSTM who were seen at our outpatient clinic. Four violinists with PBT described previously were included. During anamnesis, by mail or via telephone the following information were asked for: Age, age when starting to play the instrument and age at onset of tremor; time between starting to play the instrument and tremor onset; previous trauma; whether the professional position as before tremor was still upheld (e.g. an orchestra musician was still playing in an orchestra); responsiveness to alcohol; family history for movement disorders. Tremor frequency; side of tremor; spreading of tremor to other tasks; treatments and their efficacy. In order to distinguish between kinetic tremor and postural tremor at the instrument, the former and latter was considered type A and B tremor, respectively. However we are aware that this distinction may be debatable (Table 1). Tremor was quantified with electromyogram and accelerometer in 22 patients and the mean frequency is given for those participants. Details will be reported elsewhere.

To assess the gender distribution we applied two *χ*^2^-tests: The first assuming no gender predominance and a second one assuming a gender predominance as in musician’s dystonia [22] (f:m =1:4), if the first should reveal a significant difference.

**Table 1:** **Patients’ characteristics of all 23 patients with a task specific tremor at the instrument**

	Gender	Age (yrs)	Instrument	Age when starting to play the instrument (yrs)	Age at onset of TST (yrs)	Time until TST onset since beginning to play (yrs)	TST duration (yrs)	Tremor location	Type A tremor	Type B tremor	Spreading of tremor	Previous trauma	Medication	Botulinum toxin	Ethanol responsive	Family history for movement disorders	DBS	Tremor frequency (Hz)	Still plays in the orchestra
Pat. 1	Male	40	Sax	12	27	15	13	Wrist	l	n	y	y (overuse)	Thx	y	n	n	n	7.3	n
Pat. 2	Male	62	Violin	8	46	38	16	Wrist	r	n	y	y (car accident)	Prop, Thx, Gabap,Topi, Prim	y	y	y (WC)	n	6.4	y
Pat. 3	Male	54	Violin	12	43	31	11	Wrist	r	n	y	n	Prim, Prop	n	y	y (tremor)	n	4.9	y
Pat. 4	Male	48	Violin	4	40	36	8	Wrist	r	n	y	n	Prim, Prop	y	y	n	n	6.8	y
Pat. 5	Female	50	Violin	13	43	30	7	Wrist	r	y	n	y (overuse)	Prop, Thx	n	n	n	n	6.4	y
Pat. 6	Female	24	Violin	5	10	5	14	Wrist	r	n	n	n	Thx, Mad, Cip, Prop	n	?	n	n	6.8	Student
Pat. 7	Male	59	Violin	7	58	51	1	Wrist	r	n	n	y (finger injury)	Thx	n	n	n	n	8.3	y
Pat. 8	Male	62	Violin	7	59	52	3	Wrist	r	n	y	y (overuse)	Prop, Thx	n	?	n	n	7.1	y
Pat. 9	Male	54	Violin	10	44	34	10	Wrist	r	y	n	n	Prop	n	n	y (tremor, PD)	n	4.6	y
Pat. 10	Male	54	Guitar	11	45	34	9	Pron/Sup	r	y	y	n	Thx	n	y	n	n	6.8	Teacher
Pat. 11	Female	58	Cello	7	56	49	2	Pron/Sup	l	n	n	y (fracture radius + ulna)	Prop	n	?	n	n	6.1	y
Pat. 12	Male	63	Cello	9	61	52	2	Pron/Sup	l	n	n	n	none	n	n	n	n	5.1	Retired
Pat. 13	Male	56	Sax	10	47	37	9	Wrist	l	n	y	n	Prop	y	y	n	n	6.4	y (Big Band)
Pat. 14	Female	55	Oboe	15	50	35	5	Elbow	r	n	y	n	Prop, Prim	y	n	n	n	6.4	y
Pat. 15	Male	57	Violin	10	40	30	17	Shoulder	r	y	n	y (surgery shoulder)	Vfx, Mtz, Loraz, Thx	n	?	n	n	6.6	y
Pat. 16	Male	63	Viola	6	62	56	1	Elbow	l	y	y	n	Prop, Mad	n	n	n	n	6.4	y
Pat. 17	Male	23	Piano	6	19	13	4	Wrist	l	n	n	n	Prop	n	n	n	n	6.4	Student
Pat. 18	Male	38	Cello	5	28	23	10	Wrist	r	y	n	n*	Thx, Cip, Mad	n	n	y	n	6.8	y
Pat. 19	Male	58	Violin	9	57	48	1	Wrist	r	y	n	y (surgery rotaor cuff)	Bromazepam	n	n	y (ET)	n	7.6	y
Pat. 20	Male	55	Violin	12	54	42	1	Wrist	r	y	n	y (pain shoulder)	Prop	n	y	y (ET)	n	6.1	y
Pat. 21	Male	59	Violin	12	49	37	10	Elbow	r	y	n	n	-	n	?	y (ET)	n	7.8	Teacher
Pat. 22	Male	55	Oboe	13	54	41	1	Elbow	l	y	y	n	Prop	n	n	y (ET)	n	6.1	Self employed
Pat. 23	Male	38	Percussion	14	33	19	5	Wrist	l	n	y	n	Cip, Prop	n	n	y (PD)	n	-	y

*This patient had a dystonia that improved before tremor occured. *Abbreviations*: *yrs* years, *Pat.* patient, *DBS* deep brain stimulation, *r* right, *l* left, *y* yes, *n* no, *Prop* propranolol, *Thx* Trihexyphenidyl, *Gabap* Gabapentin, *Topi* topiramat, *Prim* primidone, *Mad* madopar, *Cip* cipralex, *Vfx* venlafaxin, *Loraz* lorazepam, *WC* writer’s cramp, *PD* Parkinson’s Disease, *ET* essential tremor.

## Results

### Epidemiology

**Figure 1:**
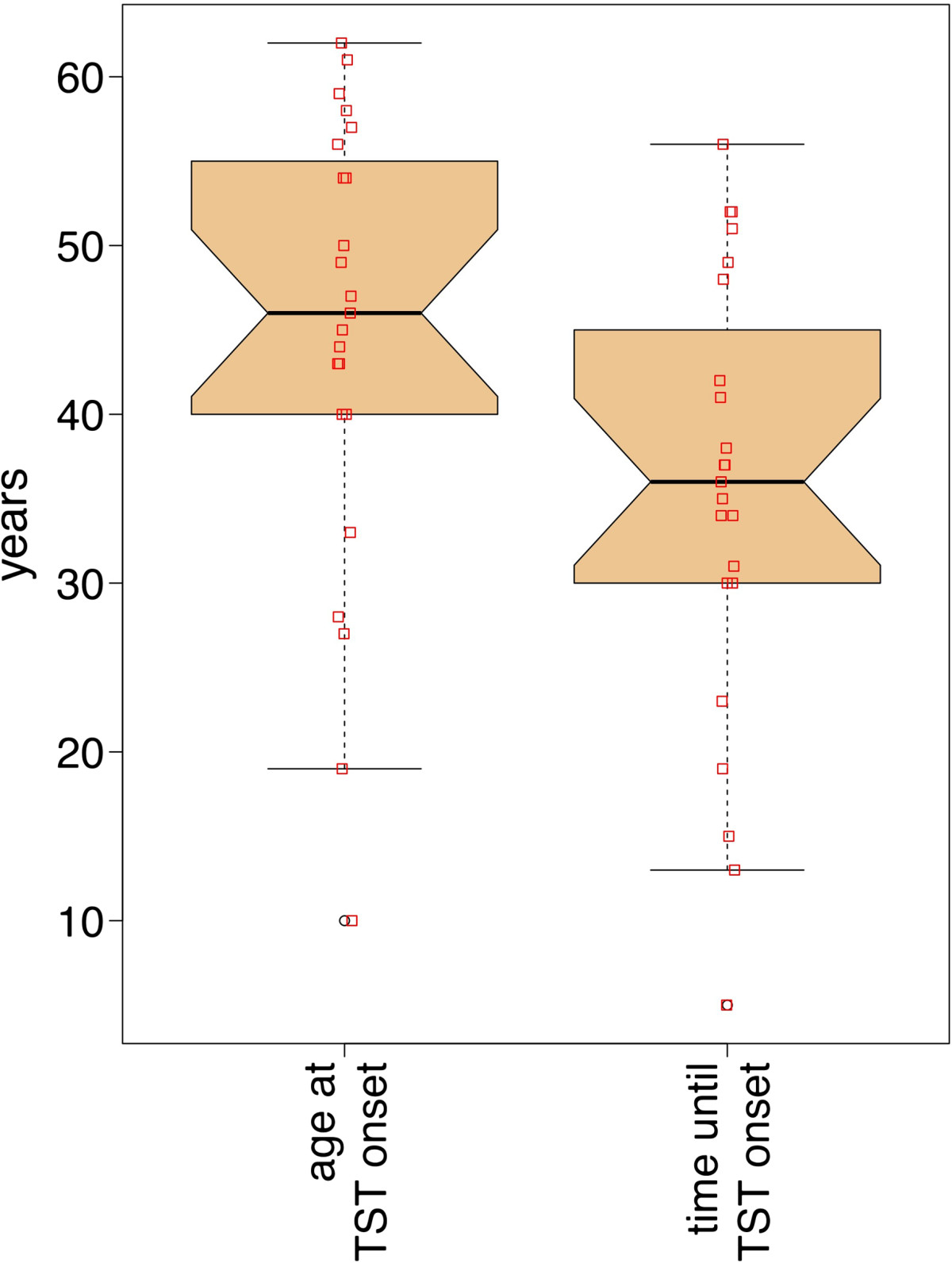
**Boxplot of the onset age of the task-specific tremor (left) and the time between starting to play the instrument and tremor.** TST = task specific tremor

Mean ± standard deviation (SD) of age of our patients was 51.5 ± 11.4 years. Tremor duration was 7.0 ± 5.1 years. Patients started their instrument at an age of 9.4 ± 3.2 years. Age at onset of the TST was 44.6 ± 13.6 years and duration between starting to play the instrument and tremor onset was 35.1 ± 13.5 years (Figure 1). Of our patients, sixteen were string instrumentalists (69.6%), four were woodwind instrumentalists (17.4%) one was a guitarist, pianist and percussionist respectively (each 4.3%). Nine patients (39%) reported a trauma or injury prior to onset of tremor: three had an overuse injury of the respective arm after intensively practicing and playing the instrument, one had an injury of the middle and ring finger and one had a fracture of the radius and ulna. Three patients had shoulder related problems: One had surgery of the shoulder, one surgery of the rotator cuff and one a pain syndrome. Nine patients (39%) had a positive family history for tremor: Two (9%) had relatives with Parkinson’s disease, of which one had another relative with a tremor disorder. One patient (4%) had a relative with writer’s cramp, four (17%) had a relative with essential tremor and one (4%) had a relative with an unknown tremor disorder. In eight of the patients (35%) a first-degree relative and in four (17%) a second-degree relative was affected. Three patients (13%) had two affected relatives. Three of the four relatives with essential tremor were first-degree relatives (parents) and one was a second degree relative (grandparents). Two patients did not recall which kind of tremor disorder their relatives were suffering from. In one patient, a first degree relative (father) was suffering from writer’s cramp. One patient had a dystonia before onset of TSTM, however at clinical examination no dystonic posturing was visible, and only tremor was discernible.

### Phenomenology

Tremor frequency was 6.5 ± 0.9 Hz. Ten patients (43%) were diagnosed as type B tremor. Fifteen patients (65.2%) had a wrist tremor, three (13.0%) had a forearm pronation supination tremor, four (17.4%) had an elbow tremor and one (4.3%) had a shoulder tremor. In eight patients (35%) tremor was at the left upper extremity. Seventeen patients (74%) were orchestra musicians (including big band), two (9%) were students, two (9%) were teachers, one (4%) was self-employed and one (4%) was retired. In all patients, tremor started while playing the instrument and remained unilateral. None of the patients developed rest tremor and no other posture than for playing the instrument was affected. In eleven patients (48%) tremor has spread to other activities. In one patient tremor spread first to shaving, in another to an instrument that was not affected at first, in two tremor appeared when holding a pen, in five when filling a glass with water, one patient had difficulties holding the telephone or typing on a computer keyboard, one had tremor when playing table-tennis. Spreading of tremor occurred between 1 and 6 years after tremor during playing the instrument. Tremor was alcohol responsive in six patients (33% of 18 patients, five patients did not drink any alcohol). In none of those patients did alcohol lead to a complete remission of tremor, therefore its effect was not rated as sufficient by any of the patients. All opposed taking alcohol before concerts. None of those patients reported a dose response curve to alcohol; however, none had consciously assessed a possible dose–response. Likewise none of those patients reported a rebound effect.

### Treatment

**Figure 2:**
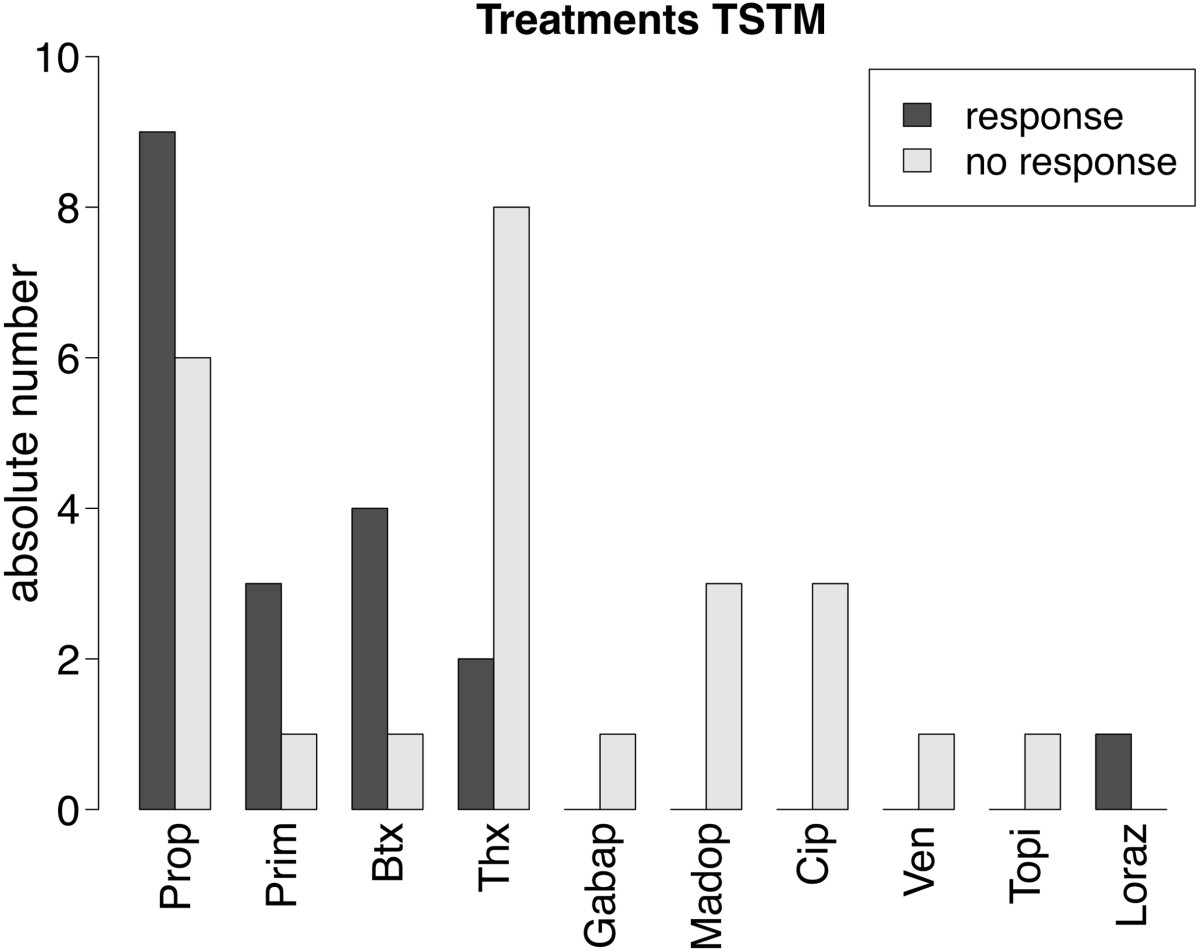
**Response to treatments given in our study in absolute numbers.** TSTM = Task specific tremor in instrumentalists; Prop = Propranolol; Prim = Primidone; Btx = Botulinumtoxin; Thx = Trihexyphenidyl; Gabap = Gabapentine; Madop = Madopar; Cip = Cipralex; Ven = Venlafaxine; Topi = Topiramate; Loraz = Lorazepam.

Propranolol was taken in dosages between 5–80 mg/day by 15 patients, of whom 9 had an improvement of tremor (60%); Trihexyphenidyl was taken by 10 patients at a dosage of 6 mg/day of whom 2 reported an improvement (20%); four patients took Primidone at 60 mg/day, of whom 3 had an improvement of tremor (75%); Madopar® at a dosage of 62,5 mg/day and Escitalopram at a dosage of 10 mg/day were taken by three patients respectively without effect; Gabapentin at a dosage of 2.4 g/day, Venlafaxin at a dosage of 150 mg/day, Topiramat at a dosage of 200 mg/day and Lorazepam at a dosage of 1 mg/day was each taken by one patient without effect. Five patients were treated with injections of Botulinumtoxin with amounts of 10–117.5 Units or 5–23.4 Units/muscle, where the amount of muscles varied between two and six. No adverse events were reported under this treatment. Four of these patients reported an improvement of tremor (80%). Treatment and response to treatment are given in Table 2 and Figure 2.

**Table 2:** **Medications prescribed**

	Prop	Thx	Prim	Madop	Gabap	Cip	Ven	Topi	Loraz	Btx*
Number of patients	15	10	4	3	1	3	1	1	1	5
Improvement	9	2	3	0	0	0	0	0	1	4
No improvement	6	8	1	3	1	3	1	1	0	1
Dosage	5-80 mg	6 mg	60 mg	62.5 mg	2.4 g	10 mg	150 mg	200 mg	1 mg	5-23.4/muscle

*Units of Dysport® were converted intor units of Botox® with the factor 3:1. Thx = Trihexyphendyl; Gabap = Gabapentine; Madop = Madopar; Prop = Propranolol; Prim = Primidone; Cip = Citalopram; Ven = Venlafaxin; Topi = Topiramate; Loraz = Lorazepam; Btx = Botulinumtoxin; resp = responder; non-resp = non-responder.

### Gender distribution

*χ*^2^-test assuming an equal gender distribution (f:m =1:1) demonstrated that *χ*^2^ = 9.8, df =1, and p =0.002. *χ*^2^-test assuming a gender distribution as in musicians dystonia (f:m =1:4) showed *χ*^2^ = 0.1, df =1, p =0.8.

A summary of epidemiology, phenomenology and treatment in this study, in PBT and in PWT is given in Table 3.

**Table 3:** **Summary of epidemiology, phenomenology and treatment in this study, in primary bowing tremor (PBT) and primary writing tremor (PWT)**

	Mean age at onset (yrs)	SD of onset age (yrs)	Sample size	Type B	Alcohol responsive	Antichol	Dosage per day	Prop	Dosage per day	Prim	Dosage per day	BTX	Dosage per day	Family history	Trauma	Tremor frequency (Hz)	Ratio f:m
**TST in musicians**																	
This paper	51.5	11.4	n = 23	10 (43%)	6/18 (33%)	2/10 (20%)	6 mg	9/15 (60%)	5-80 mg	3/4 (75%)	60 mg	4/5 (80%)	5-23.4 U/m	9 (39%)	9 (39%)	6.5 (+ − 0.9)	4:19
**PBT**																	
Lederman [2]	41	23.2	n = 5	2 (40%)	1/1 (100%)	0/1	6 mg	1/1 (100%)	20 mg	-	-	-	-	2 (40%)	-	-	3:02
**PWT**																	
Rosenbaum & Jankovic [7]	50.5	14.8	n = 10	3 (30%)	2/4 (50%)	4/5 (80%)	-	4/4 (100%)	-	-	-	-	-	3 (30%)	-	-	3:07
Bain et al. [5]	50.1	range (16–76)	n = 21	10 (48%)	7/17 (41%)	4/12 (33%)	-	4/12 (33%)	-	3/4 (75%)	62.5-125 mg	2/2 (100%)	33 U/m	7 (33%)	4 (19%)	5.5 (4.1-7.3)	1:20
Kachi et al. [6]	40.8	18.1	n = 10*	3 (30%)	3/4 (75%)	-	-	10/11 (91%)#	120-240 mg+	-	-	-	-	0	3 (30%)	5-Jun	2:08
Elble et al. [8]	37	13.9	n = 5	2 (40%)	1/4 (25%)	1/3 (33%)##	12-15 mg++	0/3 (0%)	240 mg	0/2 (0%)	750 mg	-	-	1 (20%)	-	5-Jul	1:04
Modugno et al. [12]	51.7	16.2	n = 7	1 (14%)	-	-	-	-	-	-	-	-	-	3 (43%)	-	5-Jul	1:06
Ondo & Satija [19]	47.2	18	n = 56	11 (20%)	-	-	-	31**	-	18**	-	17**	-	27 (48%)	-	-	41:15:00
Papapetropoulos & Singer [14]	48.4	15.5	n = 5	2 (40%)	0/5	1/2 (50%)	6 mg	-	-	-	-	4/4 (100%)	10-12.5 U/m	0	-	-	2:03

*One patient had golfing tremor; # in five patients propranolol was administered intravenously and not orally, of whom 4 resonded; +only orally administered propranolol; ##2xtrihexyphenidyl, 1xbenztropine (no effect); ++dosage only for trihexyphenidyl; **only the number of treated patients was reported with a mean rating of success; ## not includeing the range given by Bain et al. *Abbreviations*: *TST* Task-specific tremor, *PBT* Primary bowing tremor, *PWT* Primary writing tremor, *yrs* years, *SD* standard deviation, *Type B* type B task-specific tremor, *Antichol* anticholinergic medication, *Prop* propranolol, *Prim* primidone, *f* female, *m* male, *appr* approximately.

For the treatment and alcohol responsiveness, the numerator indicates positive response to the respective medication/alcohol whereas the denominator indicates the number of patients treated with the respective medicament/alcohol in the respective study.

## Discussion

Tremor frequency corresponds to what has been found in PWT [5, 6, 8, 12]. The main differences to PWT are firstly that we found an involvement of proximal muscles, which to our knowledge has not been reported in PWT. The necessity to hold the entire arm against gravity while playing the instruments in contrast to writing may be an explanation for this phenomenon. Secondly type B tremor occurs more often in TSTM than in PWT [5–8, 12, 14, 19]. This is interesting because it supports the notion that the tonic nature of playing the affected instruments as mentioned above seems to play a role in TSTM. No difference to PWT [5–8, 12, 14, 19] was found with regard to alcohol responsiveness, although a wide range between 0% [14] and 75% [6] exists in PWT (Table 3). Spreading of tremor to other tasks as we found in TSTM is a phenomenon described in TST and a strict task-specificity was questioned recently [5, 19, 23]. An evolution of the disorder over time [19] was discussed that may apply to TSTM, as well.

The mean onset age of TSTM is 43.5 years and does not differ to PBT [1, 2] and is only marginally lower than in PWT [5–8, 12, 14, 19] (Table 3). The high standard deviation in PBT [2] and in PWT [5–8, 12, 14, 19] reflects the great range of the age at tremor onset (Table 3). Tremor appeared on average 35 years after starting to exert the triggering task, an information to our knowledge not reported in PWT. However there is a wide range of 5 years to more than 50 years. Mean tremor-affected duration of our patients was 7.6 ± 5.2 years. In this context it is noteworthy that only one of the musicians playing in an ensemble had to stop playing because of tremor and all musicians who are primarily teaching continue to exert their profession. One musician was retired (Table 3). This is an important finding, since for most musicians tremor poses a serious threat to their professional career. Which treatments are available that may allow patients to continue performing (Table 3)? Botulinum-toxin-injection with a response rate of 80% was the most effective treatment. High response rates have been reported in PWT [5, 14, 15], where it was fond to be the second-best rated treatment after deep brain stimulation [19]. The most effective oral medication was Primidone with a response rate of 75%. Propranolol was the most often prescribed treatment and beneficial for 60% of our patients, although, as in PWT [6–8] improvement was not related to the dosage: one patient reported an improvement under 5 mg whereas 80 mg did not have any effect in another patient. As in PWT [5, 7, 8, 14], Trihexyphenidyl was beneficial for only 20% of the patients, although one patient had an almost complete remission. The effect of Lorazepam, taken by one patient was due to reduction of stress caused by the tremor. None of the other medications had any effect. Treatment in PWT and PBT are given in Table 2.

None of our patients has undergone DBS so far, although two had considered it. The main concern is the lack of experience with DBS in musicians as well as high expectations of a complete remission. Therefore a recent case report of an oboist with TST is of interest, where a marked improvement through bilateral stimulation of the ventralis intermedius nucleus was reported [24]. In PWT several reports of successful treatment with DBS of the thalamus exist [9, 11, 17].

The response to Primidone and Propranolol is suggestive of essential tremor and it remains under debate whether task-specific tremors are a form of essential tremor, of focal dystonia or an entity of its own [7, 18, 23, 25].

The high prevalence of string and woodwind instrumentalists has been reported before in a study by Lederman [21]. It has to be noted, however, that in the study by Lederman tremors other than TST were included. It is of interest that in the present study that includes only patients with TSTM, tremor is much more common in instrumentalists requiring steady (tonic) postural muscular activity (holding the bow, the oboe etc.) as in instrumentalists mainly relying on phasic and ballistic movements such as pianists and percussionists. This of course may be related to the professional consequences, in that postural tremor reduces perceived sound quality to a much higher degree as compared to tremor in phasic movements.

A recent study of five string instrumentalists with PBT consisted of three female and two male participants suggesting an female gender predominance [2], however, due to the small sample size general conclusions were difficult. Our test for gender distribution suggests a male gender predominance in TSTM that does not differ to musician’s dystonia [22] or PWT.

Of our patients, 39% reported a trauma of the affected limb prior to tremor onset, which is known from PWT, albeit at a lower prevalence (19% [5] and 30% [6]), however, we are aware that this question is subject to recall bias. Whether traumata are related to the development of TST, however, cannot be concluded from our data alone. One patient had a dystonia that had markedly improved before developing tremor and during examination at the instrument no dystonic posturing was visible and only tremor was evident. This is interesting, because dystonia preceding PWT was described by Bain et al. [5] and likewise tremor preceding dystonia has been described before [26]. A positive family history as risk factor was found in PBT [2] as well as in PWT, although in PWT, two studies reported no family history in ten [6] or five [14] patients respectively (Table 3).

## Conclusion

To our knowledge, this is the largest sample of TSTM described in detail so far. It is a heterogeneous disorder that shares many commonalities with PWT. It occurs mainly between the age of 30 and 55, appears between 21 and 48 years after starting to play the instrument and has a male gender predominance. It may spread to other tasks. Because previous trauma may precede TSTM, it is important to raise awareness for preventive measures against playing related injuries as e.g. overuse. There appears to be a genetic predisposition for TST. A variety of treatment options exist, of which most beneficial are Botulinumtoxin, Propranolol, and Primidone. Since no single beneficial medication exists, treatment of patients remains highly individual. Given the positive results of DBS in PWT and the positive case report of an oboist with TSTM, it should be discussed, whether this should be offered not only to patients that do not respond to any other medication but early in the course of the disease. However, further studies are necessary to address this question.

## Authors’ original submitted files for images

Below are the links to the authors’ original submitted files for images. Authors’ original file for figure 1 Authors’ original file for figure 2 Authors’ original file for figure 3 Authors’ original file for figure 4
