# Supplementary figures and images for: Epidemiology and treatment of 23 musicians with task specific tremor
Source: J Clin Mov Disord. 2014 Dec 4;1:5. doi: 10.1186/2054-7072-1-5 (PMC4677731; doi:10.1186/2054-7072-1-5)

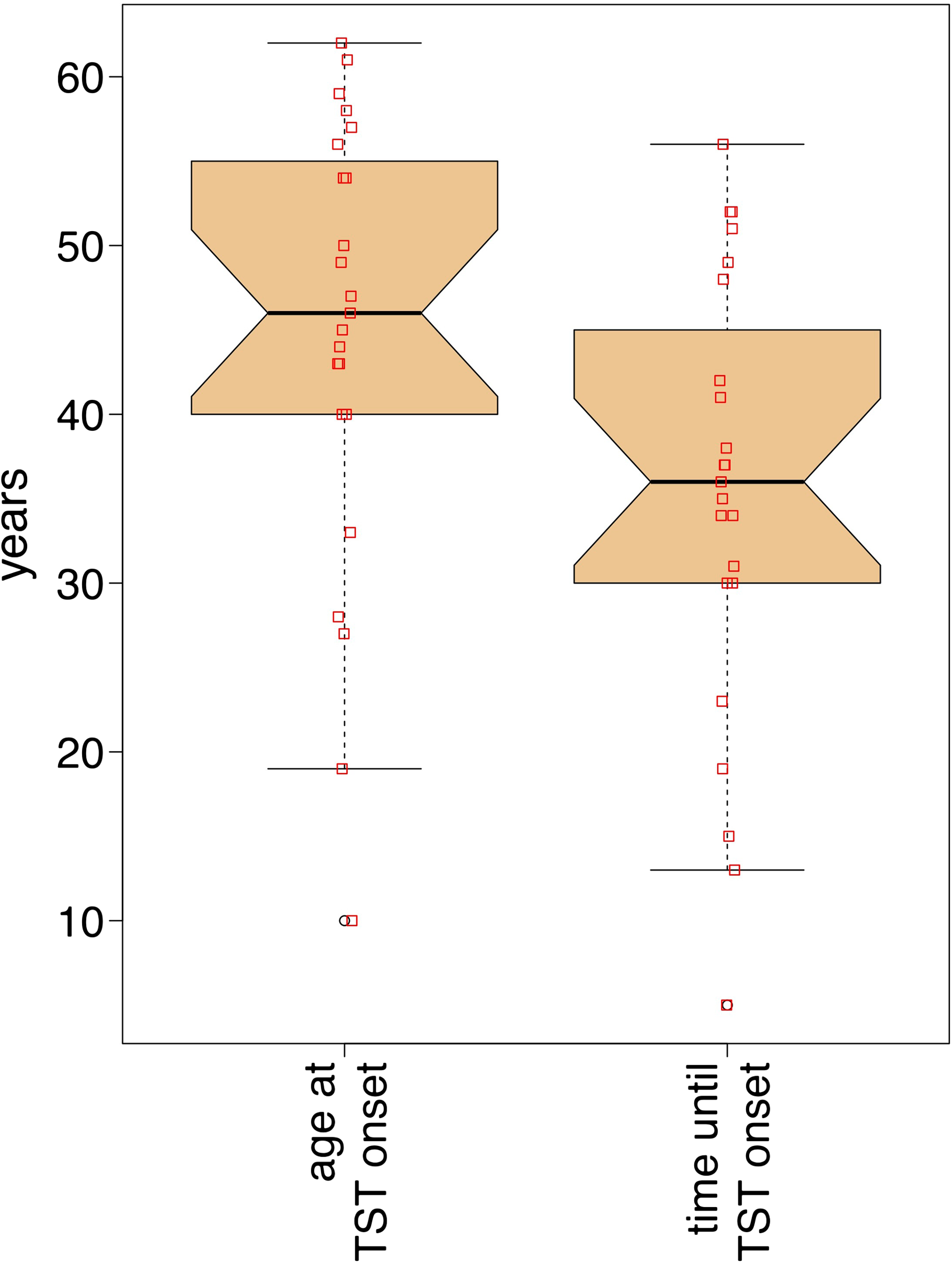

Supplement: Supplementary file 1 — Authors’ original file for figure 1 [file 40734_2014_7_MOESM1_ESM.tiff]

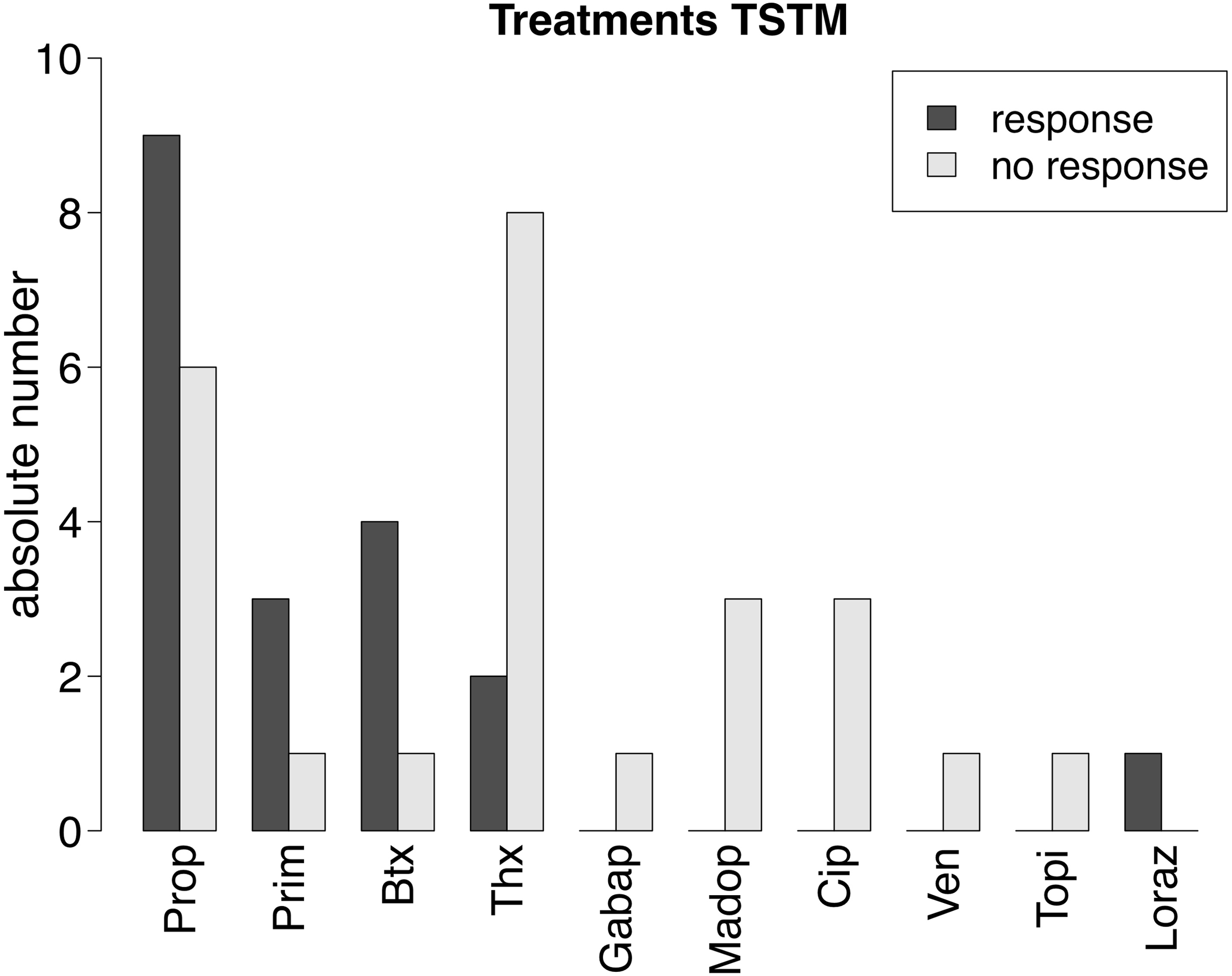

Supplement: Supplementary file 2 — Authors’ original file for figure 2 [file 40734_2014_7_MOESM2_ESM.tiff]

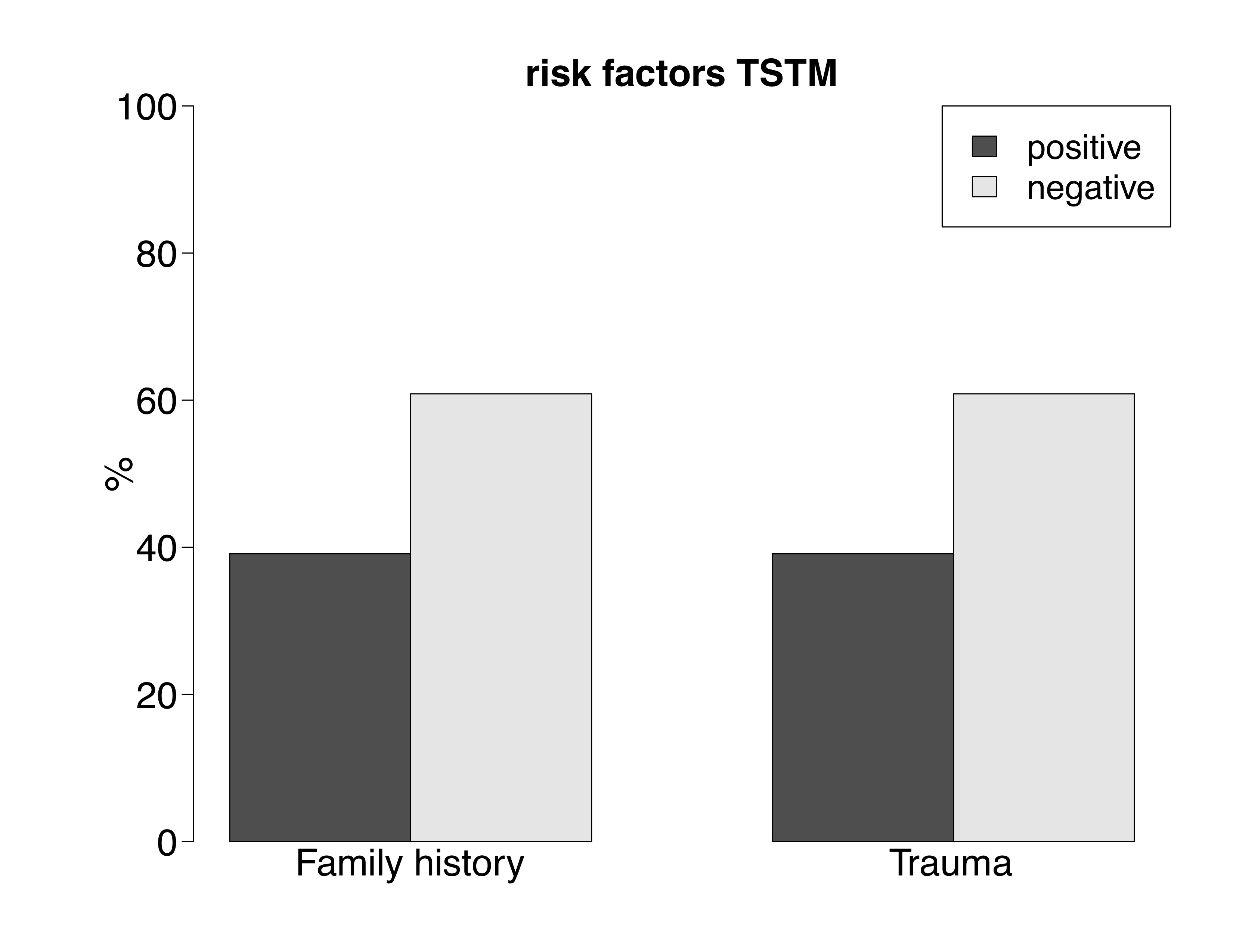

Supplement: Supplementary file 3 — Authors’ original file for figure 3 [file 40734_2014_7_MOESM3_ESM.jpeg]

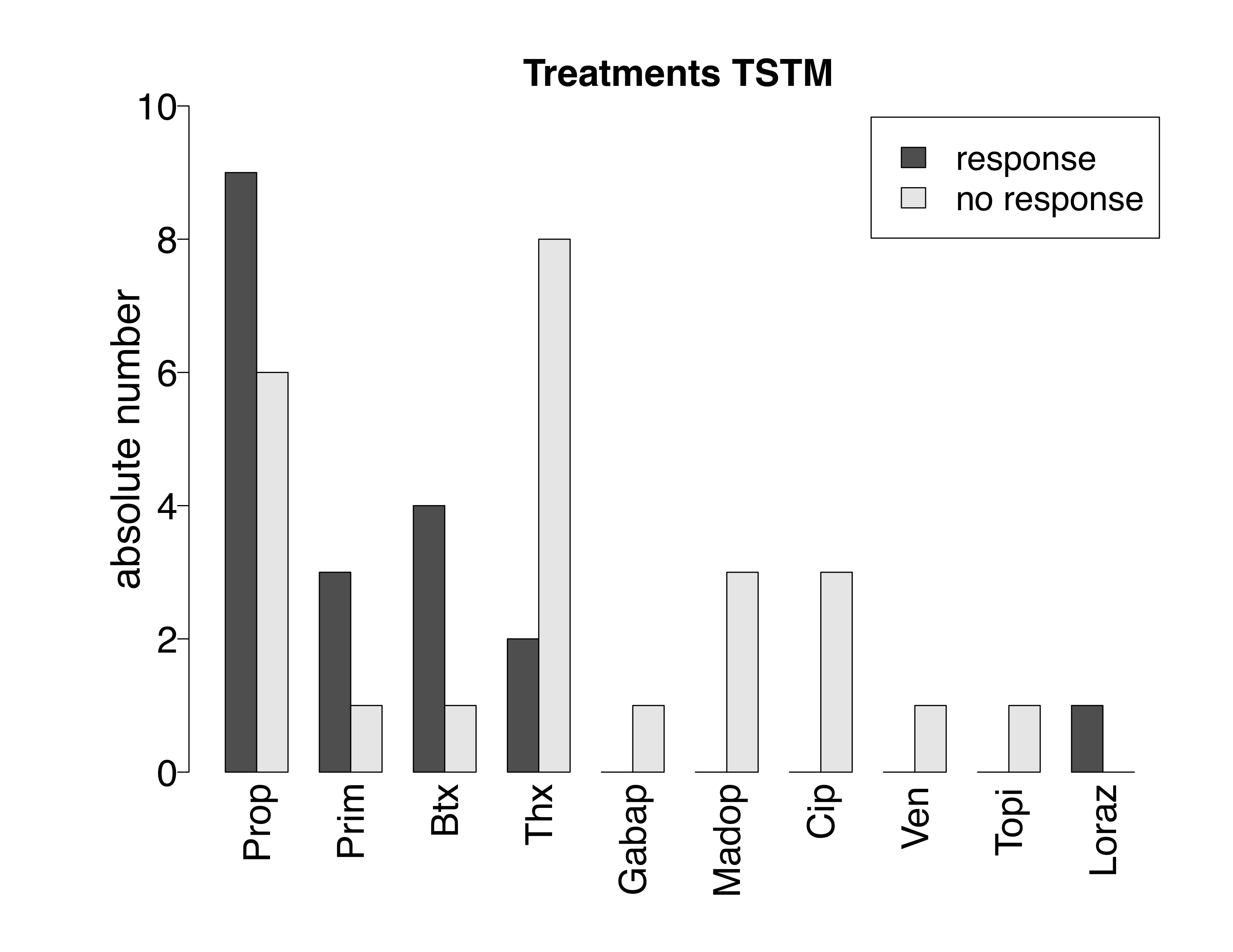

Supplement: Supplementary file 4 — Authors’ original file for figure 4 [file 40734_2014_7_MOESM4_ESM.jpeg]
